# Globally, plant‐soil feedbacks are weak predictors of plant abundance

**DOI:** 10.1002/ece3.7167

**Published:** 2021-01-27

**Authors:** Kurt O. Reinhart, Jonathan T. Bauer, Sarah McCarthy‐Neumann, Andrew S. MacDougall, José L. Hierro, Mariana C. Chiuffo, Scott A. Mangan, Johannes Heinze, Joana Bergmann, Jasmin Joshi, Richard P. Duncan, Jeff M. Diez, Paul Kardol, Gemma Rutten, Markus Fischer, Wim H. van der Putten, Thiemo Martijn Bezemer, John Klironomos

**Affiliations:** ^1^ Fort Keogh Livestock & Range Research Laboratory United States Department of Agriculture‐ Agricultural Research Service Miles City MT USA; ^2^ Department of Biology Institute for the Environment and Sustainability Miami University Oxford OH USA; ^3^ Department of Biology Alma College Alma MI USA; ^4^ Department of Integrative Biology University of Guelph Guelph ON Canada; ^5^ Laboratorio de Ecología Biogeografía y Evolución Vegetal (LEByEV) Instituto de Ciencias de la Tierra y Ambientales de La Pampa (INCITAP) Consejo Nacional de Investigaciones Científicas y Técnicas (CONICET)‐Universidad Nacional de La Pampa (UNLPam) Santa Rosa Argentina; ^6^ Departamento de Biología Facultad de Ciencias Exactas y Naturales UNLPam Santa Rosa Argentina; ^7^ Grupo de Ecología de Invasiones INIBIOMA Universidad Nacional del Comahue CONICET San Carlos de Bariloche Argentina; ^8^ Department of Biological Sciences Arkansas State University Jonesboro AR USA; ^9^ Institute of Biochemistry and Biology University of Potsdam Potsdam Germany; ^10^ Berlin‐Brandenburg Institute of Advanced Biodiversity Research (BBIB) Berlin Germany; ^11^ Leibniz Centre for Agricultural Landscape Research (ZALF) Müncheberg Germany; ^12^ Institut für Biologie Plant Ecology Freie Universität Berlin Berlin Germany; ^13^ Institute for Landscape and Open Space Eastern Switzerland University of Applied Sciences St. Gallen Switzerland; ^14^ Centre for Conservation Ecology and Genetics Institute for Applied Ecology University of Canberra Canberra ACT Australia; ^15^ Institute of Ecology and Evolution University of Oregon Eugene OR USA; ^16^ Department of Forest Ecology and Management Swedish University of Agricultural Sciences Umeå Sweden; ^17^ Institute of Plant Science University of Bern Bern Switzerland; ^18^ Laboratoire d'Ecologie Alpine (LECA) Université Grenoble Alpes UMR CNRS‐UGA‐USMB 5553 Grenoble France; ^19^ Department of Terrestrial Ecology Netherlands Institute of Ecology Wageningen The Netherlands; ^20^ Laboratory of Nematology Wageningen University Wageningen The Netherlands; ^21^ Institute of Biology Section Plant Ecology and Phytochemistry Leiden University Leiden The Netherlands; ^22^ Department of Biology University of British Columbia Kelowna BC Canada

**Keywords:** community composition, meta‐analysis, plant abundance, plant dominance, plant rarity, plant‐soil feedbacks, soil biota, species coexistence

## Abstract

Plant‐soil feedbacks (PSFs) have been shown to strongly affect plant performance under controlled conditions, and PSFs are thought to have far reaching consequences for plant population dynamics and the structuring of plant communities. However, thus far the relationship between PSF and plant species abundance in the field is not consistent. Here, we synthesize PSF experiments from tropical forests to semiarid grasslands, and test for a positive relationship between plant abundance in the field and PSFs estimated from controlled bioassays. We meta‐analyzed results from 22 PSF experiments and found an overall positive correlation (0.12 ≤ $\bar{r}$ ≤ 0.32) between plant abundance in the field and PSFs across plant functional types (herbaceous and woody plants) but also variation by plant functional type. Thus, our analysis provides quantitative support that plant abundance has a general albeit weak positive relationship with PSFs across ecosystems. Overall, our results suggest that harmful soil biota tend to accumulate around and disproportionately impact species that are rare. However, data for the herbaceous species, which are most common in the literature, had no significant abundance‐PSFs relationship. Therefore, we conclude that further work is needed within and across biomes, succession stages and plant types, both under controlled and field conditions, while separating PSF effects from other drivers (e.g., herbivory, competition, disturbance) of plant abundance to tease apart the role of soil biota in causing patterns of plant rarity versus commonness.

## Graphical abstract

Across studies, we detected a small but significant positive correlation between plant‐soil feedbacks measured during controlled experiments and plant field abundance. Plants that are rare are seemingly burdened more by the negative effects of harmful soil biota than common plants.

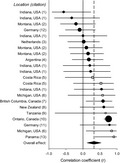

## 1 INTRODUCTION

A long‐standing challenge in ecology is to reveal which factors regulate plant abundance, coexistence, and community composition (Grilli et al., 2017). Classical ecological theory has focused on processes such as plant‐plant competition (e.g., belowground resource partitioning and aboveground light competition) and predation/herbivory to explain coexistence and assembly in natural plant communities (Palmer, 1994). Over the past two decades, attention has increasingly focused on the potential for cryptic interactions between plants and their associated soil biota to drive plant community dynamics (Bennett et al., 2017; van der Putten et al., 2013). Such interactions can facilitate or inhibit species coexistence by a process commonly referred to as plant‐soil feedback (PSF) (Bever et al., 1997). A key challenge has been to demonstrate that plant‐soil biota interactions structure plant communities in the field.

Plant‐soil feedback experiments typically compare the performance of plants in soil conditioned by conspecifics versus heterospecifics and can be used to explain conspecific facilitation or inhibition (e.g., negative frequency‐dependent effects; Bennett et al., 2017). Plant‐soil feedback experimental designs are based on the observation that individual plant species often culture divergent soil communities (Lou et al., 2014; Merges et al., 2020), and key soil biota exhibit some degree of host‐specificity (Benítez et al., 2013). Plant‐soil feedbacks are generated when (a) the soil biota that accumulate in the root zone of one plant species differ in abundance and composition from the soil biota associated with other plant species, and (b) shifts in key soil biota differentially affect the performance of recruiting plants (Bever, 1994). Negative PSF may stabilize species coexistence if a plant influences its soil biota in a way that inhibits conspecifics more than heterospecifics, thereby preventing individual plant species from dominating the community (Crawford et al., 2019). Positive PSFs occur when soil influenced by conspecifics has positive effects (Bennett et al., 2017; Dickie et al., 2014) and may contribute to clumped distributions and even monodominance. Plant‐soil feedback may also result from changes to available nutrients and nutrient pools (Ehrenfeld et al., 2005); for example, a plant species alters the availability of a nutrient(s) which then differentially impacts the performance of conspecific versus heterospecific plants in the affected soil.

To help uncover the importance of PSF to plant community assembly, several studies have used PSFs as a predictor of plant abundance and demographic patterns (Klironomos, 2002; MacDougall et al., 2011; Mangan et al., 2010; McCarthy‐Neumann & Ibáñez, 2013; Rutten et al., 2016). For example in a recent study of 55 temperate tree species, it was shown that a significant fraction (12%) of the variation in regional estimates of conspecific inhibition was explained by regional estimates of PSF (Bennett et al., 2017). There is also evidence that conspecific inhibition, caused by soil biota, is most pronounced in low density populations (Xu et al., 2015). Negative PSF are one potential driver of plant rarity and community evenness, and simulation models provide support that conspecific inhibition (e.g., negative PSF) may contribute to plant rarity, coexistence, and explain patterns in plant relative abundance (Chisholm & Muller‐Landau, 2011; Mangan et al., 2010). In contrast, other empirical studies reported negative density‐dependence that was greater for abundant than rare species (LaManna et al., 2016; Liu et al., 2015; Zhu et al., 2015), a negative correlation between abundance and plant‐soil biota effects (Maron et al., 2016), and no appreciable abundance‐PSF correlation (Bauer et al., 2015; Reinhart, 2012). Others have shown that all dominant species in a community exhibit negative PSF (Chiuffo et al., 2015; Fitzsimons & Miller, 2010; Liu et al., 2015; Olff et al., 2000; Petermann et al., 2008), which also indicates no positive abundance‐PSF correlation.

Of particular importance is the ability to summarize this conflicting information and to move beyond the idiosyncrasies of individual studies (e.g., site properties, growth conditions) to investigate the generality of the correlation between plant abundance and PSF. Here, we used meta‐analyses to test whether there is empirical evidence to support the hypothesis that the field abundance of plants is, on average, positively correlated with estimates of PSF. Meta‐analysis is an important tool in ecology because of its capacity to find general trends, even when individual studies are too small to detect such a relationship (Koricheva & Gurevitch, 2014; Koricheva et al., 2013). We also tested for this general relationship separately for herbaceous plant species only—the most prevalent plant functional type in the dataset. Differences among plant functional types in the abundance‐PSF relationship are likely because of divergence in PSF due to differences in life histories, abiotic environments (McCarthy‐Neumann & Kobe, 2008; Rutten & Gómez‐Aparicio, 2018), and/or key methodological differences between studies, especially of woody versus herbaceous taxa (e.g., Rinella & Reinhart, 2018). For example, PSF experiments for tree species may have larger impacts on soil biota, because they typically utilize field conditioned soil which has likely developed after a decade or more of association with the tree, than experiments with herbaceous taxa which mostly rely on soil conditioned in short‐term glasshouse experiments with plants propagated in pots (Kulmatiski & Kardol, 2008). Our analyses provide a first approximation of the global average relationship between plant field abundance and PSF.

## 2 METHODS

### 2.1 Literature search

Our aim was to perform a systematic search of the literature to identify PSF studies that included field abundance measurements for each plant species to be used in our meta‐analyses. All studies were identified using either a literature search, examining lists of articles that cite related studies, co‐authors’ knowledge, and by obtaining unpublished data. We used the ISI Web of Knowledge for a 30‐year period (1986–2016) to identify relevant literature with a title search: (plant* soil* feedback*) OR (soil* feedback* experiment) on August 9, 2016. Our intent was to identify studies with measures of plant performance when grown in pots with soil conditioned by conspecifics and in pots with soil conditioned by heterospecifics. In addition, the studies should contain measures of field abundance for each plant species. In several cases, plant abundance data either happened to be available though not with the published PSF data (Giesen, 2006; McCarthy‐Neumann & Kobe, 2010), was unpublished, or was subsequently collected (McCarthy‐Neumann & Ibáñez, 2012). Researchers with relevant data were invited to collaborate. Collaboration facilitated data sharing and standardization, discovery and inclusion of unpublished data, and discovery of relevant studies not identified by the literature search.

**FIGURE 1:**
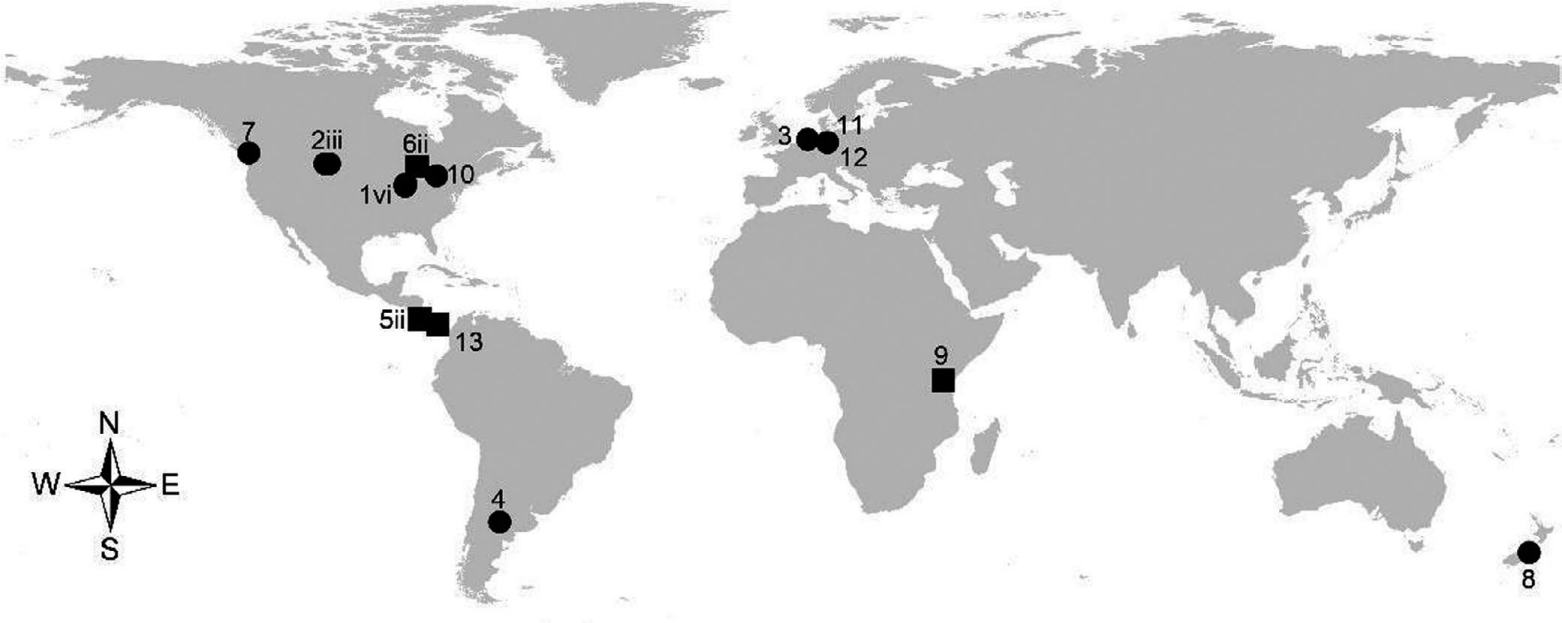
Location of experiments used in a meta‐analysis of correlation coefficients of plant field abundance and plant‐soil feedback (PSF). Location symbols distinguish experiments by plant functional types: herbaceous (circle symbols) and woody (square symbols). Numbers corresponding with symbols indicate relevant citations for PSF experiments. Studies with two or more experiments are denoted with lower case Roman numerals (ii = 2, iii = 3, vi = 6). Citation numbers are defined in Figure 2

Our search resulted in broadly distributed studies from a diversity of ecosystems ranging from tropical forests to semiarid grasslands on different continents (Figure 1). We obtained data for 16 experiments with herbaceous species, mostly from temperate grasslands, and for six experiments with woody species from savannas to tropical and temperate forests. Divergent methods were typical for herbaceous versus woody species (Table A1). For four of 13 studies, ecologically distinct subsets of data were treated as independent experiments (Figure 1), because PSF were estimated for either separate sites or light environments (see “other factors” of Table A1). Across PSF studies, the experimental designs essentially compared plant performance (e.g., total biomass, survival) when grown in soil conditioned by conspecific versus heterospecific plant species. Aboveground plant biomass was the most common measure of plant performance in PSF experiments, but some studies included: plant growth rate, survival, and integrated measures that adjusted growth or biomass for plant mortality. Studies of herbaceous species mostly assayed the effects of soil conditioned in pots (see 2‐ or 3‐stage experiments in Table A1). Studies of woody species mostly assayed the effects of soil conditioned in the field (i.e., collected near adult conspecific versus heterospecific trees; see soil inocula experiments in Table A1) (e.g., Rinella & Reinhart, 2018). Studies also varied in the types of measures of field abundance (e.g., stem density, stem basal area, % cover, total biomass) (Tables A1 and A2). While these sources of heterogeneity are not preferred, they are also not uncommon (Kulmatiski et al., 2008; Lekberg et al., 2018). Our dataset included measures of PSFs based only on plant performance in soil conditioned by conspecifics versus heterospecifics. Related meta‐analyses tend to rely on more heterogeneous datasets, for example, estimates of PSF based on plant performance in two soil conditioning treatments: "self" (i.e., soil conditioned by conspecifics or from an area with varying abundances of conspecifics) and "nonself" (soil conditioned by conspecifics then *sterilized* or by heterospecifics) (Kulmatiski et al., 2008). The final dataset included 281 paired measures of PSF and abundance with between four to 61 taxa per experiment (average = 12.8).

### 2.2 Data standardization

We used the raw data to compute a standardized estimate of PSF across studies based on natural log response ratio. PSF = ln(X_C_/X_H_), where X_C_ is the mean plant performance (e.g., plant dry weight) when grown in pots inoculated with soil conditioned by conspecifics and X_H_ is the mean plant response variable of plants grown in pots inoculated with soil conditioned by heterospecifics (see Table A1 for details on heterospecific treatment portion). A negative PSF suggests conspecific inhibition, and a positive PSF suggests conspecific facilitation. The most commonly used approach was to calculate PSF based on mean plant dry weights (*n* = 265); however, additional calculations were also made on alternative metrics of plant performance (e.g., plant survival and plant growth rate) depending on data availability.

### 2.3 Meta‐analysis

We used meta‐analyses to synthesize correlations (Stein et al., 2014) between plant field abundance and PSF for 22 PSF experiments across plant functional types. Species‐specific values of the two variables (i.e., PSF [log response ratio] and mean abundance) were treated as individual observations in these analyses. For each experiment, we computed the Pearson correlation coefficient (*r*) between all paired combinations of metrics of PSF (e.g., based on biomass, survival) and field abundance (e.g., stem density, basal area, % cover). To provide a *conservative test* of our null hypothesis that PSF was unrelated to field abundance, we selected the data pairs yielding the largest positive correlation coefficients. This ensured the most optimistic scenario for detecting an overall positive association between plant abundance and PSF, making a result of “no correlative association” fairly conclusive. Less conservative tests were performed using the average *r* per experiment which helped to account for publication bias (i.e., reporting of most statistically significant results [*α* = 0.05]) (Table A2). Publication bias should also have been minimal since datasets with PSFs at the community‐level can be used to address other ecologically meaningful questions as evidenced by the publishing of studies with no appreciable association between field abundance and PSF (Bauer et al., 2015; Reinhart, 2012). Furthermore, the dataset includes cases where PSF data were either unpublished or published, were used to address divergent hypotheses, and may not have been paired with plant field abundance data (Table A1).

**FIGURE 2:**
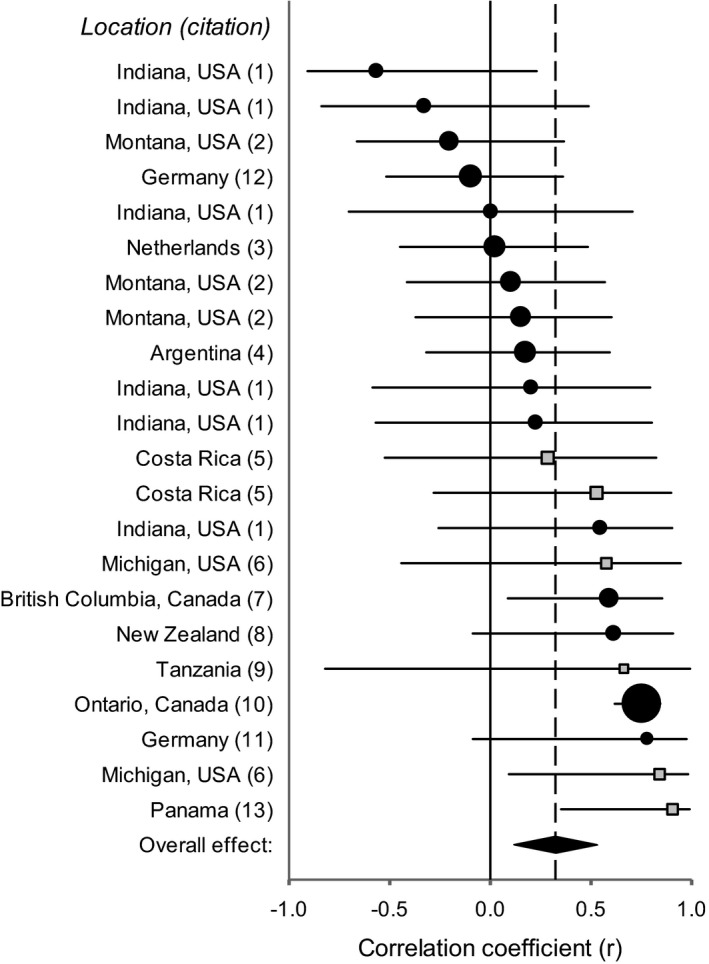
Effect sizes for the correlation between plant abundance in the field and plant‐soil feedback (PSF) for herbaceous (black circles) and woody (gray squares) plant species for many experiments. Graph depicts the results for one of eight analyses (see = $\bar{r}$ 0.32 in Table 1). Study information (location and citation) is provided to the left of the figure, the center of each symbol indicates the effect size (correlation coefficient *r*, *x*‐axis) and the whiskers indicate lower and upper 95% confidence intervals. Circle and square symbol sizes reflect the weighting (i.e., number of species per correlation coefficient) for each experiment in the analysis. The overall effect (pooled weighted correlation coefficient, $\bar{r}$) is indicated by the diamond symbol at the bottom of the plot, where $\bar{r}$= 0.32 (0.10; 0.51) and *p* = .0050. 1 = (Bauer et al., 2015), 2 = (Reinhart, 2012), 3 = (Giesen, 2006), 4 = (Chiuffo et al., 2015), 5 = (McCarthy‐Neumann & Kobe, 2010), 6 = (McCarthy‐Neumann & Ibáñez, 2012), 7 = (MacDougall et al., 2011), 8 = (Diez et al., 2010), 9 = (Rutten et al., 2016), 10 = (Klironomos, 2002), 11 = (Johannes Heinze, Joana Bergmann, and Jasmin Joshi, *unpublished*), 12 = Heinze et al., 2020), 13 = (Mangan et al., 2010)

The Pearson correlation coefficients (*r*) for individual experiments were used as effect sizes in meta‐analyses (Schulze, 2004) to obtain weighted mean correlation coefficients ($\bar{r}$) and 95% confidence intervals. Due to computational limitations, correlations based on fewer than four species were not utilized. We used a random‐effects model (instead of a less conservative, fixed‐effects model) based on Fisher's *r*‐to‐z transformation (Laliberté et al., 2010). This transformation is normalizing and variance stabilizing so that the variance depends only on sample size. Results for random‐effect models provide results with greater generality. To help prevent studies with lower power from biasing results, individual effect sizes were weighted by the inverse of their variance (e.g., Borenstein et al., 2009; Koricheva et al., 2013). The meta‐analyses of correlation coefficients was implemented with the “metacor” function in the “meta” package (Schwarzer, 2007) in R version 3.6.1 (R Development Core Team, 2011) with the DerSimonian‐Laird method to estimate the between‐study variance (DerSimonian & Laird, 1986), and presented results were back‐transformed. Because of the size of our dataset, we were able to robustly test for a general correlation between plant abundance and PSF (Field, 2001) but not the importance of other explanatory factors, such as PSF experimental design and ecosystem type (Stein et al., 2014). Therefore, separate meta‐analyses were performed for the herbaceous data subset (*n* = 16). There were too few data for the woody taxa subset (*n* = 6) to justify a separate meta‐analysis (Field, 2001). To help prevent the study with the most power (i.e., greatest number of species per experiment) from influencing results, we excluded the most influential study (i.e., study #10 of Figure 2) from the datasets (full [*n* = 21] or herbaceous only [*n* = 15]) and repeated each analysis.

## 3 RESULTS

**FIGURE A1:**
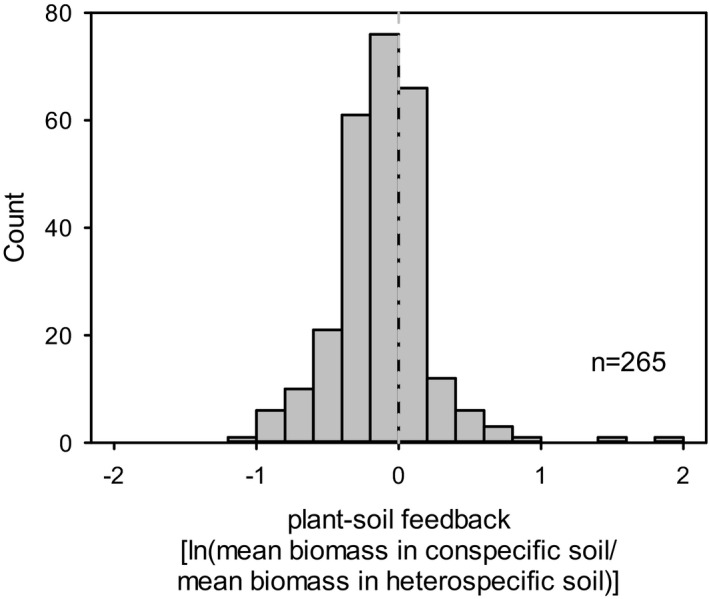
Distribution of plant–soil feedback (PSF) estimates for individual species from 21 separate PSF experiments. Prevalence of negative PSF values suggests plants often grow better in soil conditioned by heterospecifics than conspecifics

Plant biomass in soil conditioned by conspecifics was on average 11.1% lower than plants grown in soil conditioned by heterospecifics (Figure A1). Across 265 PSF measures, negative PSFs predominated and confidence intervals for the average PSF (i.e., average log response ratio = −0.118) did not overlap zero (lower 95% normal‐based confidence interval = −0.157, upper confidence interval = −0.079). This was also true for both taxa classified as herbaceous (average PSF = −0.117 [−0.158, −0.075]; *n* = 243) and woody (average PSF = −0.131 [−0.213, −0.050]; *n* = 22).

For the dataset with herbaceous and woody taxa, the pooled weighted correlation coefficient ($\bar{r}$) for plant field abundance and PSF ranged from 0.12 to 0.32. Most $\bar{r}$ differed significantly from zero (0.005 ≤ *p *≤ 0.106), and most confidence intervals did not overlap zero (Table 1, Figure 2). The main exception was $\bar{r}$ for the dataset with liberal estimates of *r* and that excluded the most influential study (*p* = 0.106). In most tests, we found evidence for a small general positive correlation ($\bar{r}$≤ 0.32) between plant abundance and PSF. However, this result depended on plant functional type. Specifically, $\bar{r}$ for the herbaceous dataset (*r* for 16 of 22 experiments) ranged from 0.07 to 0.23 and did not differ significantly from zero (0.089 ≤ *p *≤ 0.415). Confidence intervals overlapped zero (Table 1) indicating that there was no general correlation between abundance of herbaceous taxa and PSF. This finding was insensitive to the type of correlation coefficient per experiment and exclusion of the most influential study (Table 1).

**TABLE 1:** Meta‐analyses of correlations between plant abundance in the field and plant‐soil feedback measured in controlled experiments

Null test method	Most influential study	Herbaceous and woody functional types	Herbaceous functional type
Conservative^a^	Present	**0.323** (0.101; 0.515)^c^	0.228 (−0.036, 0.463)^c^
	Absent	**0.237** (0.054, 0.405)	0.134 (−0.047, 0.306)
Liberal^b^	Present	**0.241** (0.017, 0.443)^c^	0.174 (−0.091, 0.415)^c^
	Absent	0.123 (−0.028, 0.281)	0.070 (−0.099, 0.235)

Note

Pooled effect sizes (mean correlation coefficient, $\bar{r}$) and confidence intervals (95% CI in brackets) are reported for two different methods for testing the null hypothesis of no correlation, presence of the most influential study (i.e., study #10 of Figure 2), and whether analyses were of herbaceous and woody (sample size = 21–22) or only herbaceous studies (sample size = 15–16). Significant nonzero correlations (i.e., effect sizes) are in bold and are based on 95% confidence intervals.

^a^ The largest positive correlation coefficient (*r*) per experiment and conservative test of the null hypothesis.

^b^ Average *r* per experiment.

^c^ significant (*α* = 0.05) heterogeneity.

## 4 DISCUSSION

A challenge is to link cryptic interactions belowground to plant population and community dynamics. Such a link has been suggested by correlative associations between plant abundance in the field and PSF measured in controlled experiments. While synthesizing the abundance‐PSF relationships of 22 comparisons, we found that negative PSFs were a general, albeit weak, putative driver of plant rarity, with rarer plants seemingly burdened more by the accumulation of harmful soil biota. A positive abundance‐PSF relationship is consistent with demographic patterns that suggest that rare species are more sensitive than abundant species to enemies and/or intraspecific competition (Chisholm & Muller‐Landau, 2011; Yenni et al., 2017) but see Rovere and Fox (2019). Additionally, there is some evidence showing that rare plant species are rare, because they are more sensitive to soil‐borne enemies (Marden et al., 2017; Xu et al., 2015).

### 4.1 Unifying concepts

Perspectives vary on expected abundance‐PSF relationships. On one hand, negative PSFs can disproportionately harm rarer species, presumably because they either have greater pest loads or are more sensitive to pests (Marden et al., 2017; Xu et al., 2015), which likely relates to the stable coexistence of rare species (see Rabinowitz et al., 1984). On the other, negative PSFs can act in a negative frequency‐dependent manner by disproportionately harming more abundant species as their population size increases, which should also help maintain species coexistence (LaManna et al., 2016; Maron et al., 2016). For example, several studies indicated that conspecific inhibition was greater for abundant species (Bachelot et al., 2015; LaManna et al., 2016; Zhu et al., 2015).

### 4.2 Unifying methods

Meta‐analysis guides urge weighting effect sizes by metrics of study power and quality to prevent low power (or quality) studies from biasing results (e.g., Koricheva et al., 2013; Spake & Doncaster, 2017). Here tests accounted for study power (i.e., number of species per experiment) but not quality, which is difficult to categorize. If we are to understand the impact of PSFs on plant communities, then perhaps we need to address a more fundamental concern. Specifically, common approaches used to estimate PSFs (e.g., glasshouse experiments) may not reliably measure PSFs occurring in nature (e.g., Forero et al., 2019; Kulmatiski & Kardol, 2008; Peacher & Meiners, 2020). To correctly understand whether PSFs shape plant communities, PSF estimates must measure interactions in nature as accurately as possible (e.g., Peacher & Meiners, 2020; Smith‐Ramesh & Reynolds, 2017). Included studies contained appreciable heterogeneity (e.g., biomes, species pools, methodological details [Table A1]), but our dataset was too limited to control for this variation with moderator variables, especially when variables can be created ad infinitum. Even with the best imagined PSF methods, an overall positive abundance‐PSF relationship may be difficult to detect partly because plant populations and PSFs are dynamic and not necessarily in sync when (or where) measurements (or soil inocula) are collected (Chung et al., 2019). Most PSF studies rely on snapshot estimates of plant abundance and PSF (van der Stoel et al., 2002). If PSFs are dynamic and affecting unique combinations of rare and abundant species per plant community (or per unit time or space), then the most likely outcome may be detecting either no or a weak general positive abundance‐PSF relationship across communities. Moreover, abundance‐PSF relationships are likely to be affected by the generation time and life history traits of the life form(s) studied and might explain differences between herbaceous and woody vegetation.

### 4.3 Future directions

While our aim was to conduct a global synthesis, our analysis was based on studies from few geographical sites. Our main finding was a relatively weak overall positive relationship between PSF and plant abundance that was sensitive to the composition of pooled experiments (i.e., influenced by the study with the greatest power and inclusion of data for woody species). Here we list the three most urgent research foci which should help improve our understanding of abundance‐PSF relationships, and under which circumstances these relationships are likely to diverge.

1. Additional empirical tests are needed, especially in under‐represented regions (Figure 1) and for woody species or a range of functional groups within a community. Additional factors such as herbivory, plant‐plant competition, and disturbance are simultaneously affecting each plant community and may either interact with PSF or have larger effects on plant populations than PSFs (e.g., Heinze et al., 2020; Lekberg et al., 2018; Veen et al., 2014). At the same time, environmental conditions, including temperature, light, nutrients and water, might influence plants and their soil communities independently (Rinella & Reinhart, 2018). Therefore, we need more repeated PSF assessments of plant communities under changed conditions or gradients (McCarthy‐Neumann & Kobe, 2008; Rutten & Gómez‐Aparicio, 2018; Smith‐Ramesh & Reynolds, 2017).
2. Further, if a given (glasshouse or field) bioassay is to accurately estimate PSFs in nature, then it should also use the most reliable method(s) (e.g., Peacher & Meiners, 2020; Smith‐Ramesh & Reynolds, 2017). One promising advance is to replace glasshouse bioassays with field bioassays or to include complementary field experiments (Heinen et al., 2020; Smith‐Ramesh & Reynolds, 2017). Field bioassays may include well‐designed reciprocal transplants of soil cores (e.g., with or without mesh cylinders that exclude roots or roots and fungal hyphae) and seedlings (Chung et al., 2019; Reed & Martiny, 2007; Yelenik & Levine, 2011). Complementary field (or pot) experiments may include selective biocide (or other) treatments to help assess the importance of key soil biota (Bagchi et al., 2014; Bell et al., 2006; Maron et al., 2011).
3. Ecological genomics and/or other techniques that identify the primary microbes driving variation in plant performance may also help link variation in plant community structure to soil biota (Lou et al., 2014; Marden et al., 2017; Merges et al., 2020). More research using innovative, robust, and complementary research methods will help to better resolve the extent to which PSFs structure plant communities.

## CONFLICT OF INTEREST

None declared.

## AUTHOR CONTRIBUTIONS

**Kurt O. Reinhart:** Conceptualization (lead); data curation (equal); formal analysis (lead); writing–original draft (lead); writing–review and editing (lead). **Jonathan T. Bauer:** Data curation (equal); writing–review and editing (supporting). **Sarah McCarthy‐Neumann:** Data curation (equal); writing–original draft (supporting); writing–review and editing (supporting). **Andrew S. Macdougall:** Data curation (equal); writing–original draft (supporting); writing–review and editing (supporting). **José L. Hierro:** Data curation (equal); writing–review and editing (supporting). **Mariana C. Chiuffo:** Data curation (equal); writing–review and editing (supporting). **Scott A. Mangan:** Data curation (equal); writing–original draft (supporting); writing–review and editing (supporting). **Johannes Heinze:** Data curation (equal); writing–review and editing (supporting). **Joana Bergmann:** Data curation (equal); writing–review and editing (supporting). **Jasmin Joshi:** Data curation (equal); writing–review and editing (supporting). **Richard P. Duncan:** Data curation (equal); writing–review and editing (supporting). **Jeff M. Diaz:** Data curation (equal); writing–review and editing (supporting). **Paul Kardol:** Data curation (equal); writing–original draft (supporting); writing–review and editing (supporting). **Gemma Rutten:** Data curation (equal); writing–review and editing (supporting). **Markus Fischer:** Data curation (equal); writing–review and editing (supporting). **Wim van der Putten:** Conceptualization (supporting); writing–original draft (supporting); writing–review and editing (supporting). **T. Martijn Bezemer:** Writing–original draft (supporting); writing–review and editing (supporting). **John Klironomos:** Data curation (equal); writing–original draft (supporting); writing–review and editing (supporting).

## Data Availability

All data and R codes are available in Dryad https://doi.org/10.5061/dryad.3j9kd51gt.

## APPENDIX 1

**TABLE A1:** Summary of studies quantifying both plant–soil feedbacks and abundance of plants in the field

Code # (from Figure 2)	System & Location	Type of feedback experiment(s)	Heterospecific treatment	Other factors	Number of species	Plant response variables	Measures of plant abundance in the field
1vi	Tallgrass prairie, Indiana, USA	2‐stage feedback experiments	3 bioassays per species, 8 species	6 sites	8 herbaceous species per site	total biomass	percent cover
2iii	Mixed‐grass prairie, Montana, USA	3‐stage feedback experiments	1 bioassay per species, 10 species	3 sites	14–16 herbaceous species per site	total biomass	frequency (counts × m^−2^) and biomass (g × m^−2^)
3	Semi‐natural grassland, The Netherlands	2‐stage feedback experiment	1 bioassay per polyculture, 5 polycultures of 3 species		18 herbaceous species	total biomass	percent cover (relevé)
4	Prosopis woodland, Argentina	2‐stage feedback experiment	1 bioassay per species, 8 species		18 herbaceous species	aboveground biomass	percent cover
5ii^b^	Tropical forest, Costa Rica	2‐stage feedback experiments	1 bioassay per species, 5 species	low versus high light treatments	6 woody species per level of light	total biomass and biomass corrected for mortality	adult basal area (m^2^ × ha^−1^ of trees ≥ 10 cm DBH) and adult stem density (number of stems [≥10 cm DBH] × ha^−1^)
6ii^b^	Temperate forest, Michigan, USA	Soil inocula experiments	1 bioassay per species, 2–6 species	low versus high light treatments	8 woody species per level of light	growth rate, survival, and growth rate corrected for mortality	adult basal area (m^2^ × ha^−1^ of trees ≥ 10 cm DBH)^a^ and adult stem density (number of stems ≥ 10 cm DBH] × ha^−1^)
7	Invaded oak savannah, British Columbia, Canada	2‐stage feedback experiment	1 bioassay per sample, 20 random samples from a pool of samples, pool of 10 pots per species and 13 species	native versus non‐native status	14 herbaceous species	total biomass	percent cover and % presence/absence across 160 quadrats
8	Grassland, New Zealand	3‐stage feedback experiment	1 bioassay per species, 9 species		12 herbaceous species	total biomass	% presence/absence across landscape plots, local dominance scores
9	Savannah, Tanzania	Soil inocula experiment	3 bioassays per species, 3 trees per species, 4 species		4 woody species	total biomass	adult tree density (number of stems [≥10 cm DBH] × ha^−1^
10	Old field, Ontario, Canada	3‐stage feedback experiment	1 bioassay per species, 10 species		61 herbaceous species	total biomass	% presence/absence across 100 quadrants
11	Semi‐natural grasslands, Germany	2‐stage feedback experiment	1 bioassay per species, 6 species		6 herbaceous species	total biomass	percent cover
12	Semi‐natural grasslands, Germany	Soil inocula experiment	10 bioassays of a mixture, 1 mixture of 19 species		20 herbaceous species	total biomass	percent cover
13	Tropical forest, Panama	Soil inocula experiment	8 bioassays per species, 5 species		6 woody species	total biomass	adult basal area (m^2^ × ha^−1^ of trees ≥ 10 cm DBH) and adult stem density (number of stems [≥10 cm DBH] × ha^−1^)

^a^ Adult local density and basal area was determined by taking an inventory during June 2012 of presence and diameter at breast height (DBH) for all living individual trees ≥ 10 cm DBH in ten 20 × 50 m plots randomly located throughout the study area in Horner Woods.

^b^ In two studies, light level treatments were used to construct factorial experiments. Since the different light levels were analogous to PSF in different abiotic environments, the data were handled as separate experiments.

**TABLE A2:** Database of correlation coefficients (*r*) used (a, b) to test for a general relationship between plant abundance in the field and plant–soil feedbacks

Code # (from Table A1 & Figure 2)	System & Location	Study information	Correlation coefficients (*r*)
1vi	Tallgrass prairie, Indiana, USA	Site coding: i, ii, iii, iv, v, vi	*r*_i_ = 0.202^a^, ^b^; *r*_ii_ = −0.567^a^, ^b^; *r*_iii_ = −0.330^a^, ^b^; *r*_iv_ = 0.003^a^, ^b^; *r*_v_ = 0.225^a^, ^b^; *r*_vi_ = 0.546^a^, ^b^
2iii	Mixed‐grass prairie, Montana, USA	Site coding: i, ii, iii; Abundance coding: _a_ (frequency), _b_ (biomass)	*r*_ia_ = −0.205^a^, *r*_ib_ = −0.273, *r*_i_ = −0.239^b^; *r*_iia_ = 0.102^a^, *r*_iib_ = 0.013, *r*_ii_ = 0.058^b^; *r*_iiia_ = 0.152^a^, *r*_iiib_ = 0.074, *r*_iii_ = 0.113^b^
3	Semi‐natural grassland, The Netherlands	NA	*r* = 0.023^a^, ^b^
4	Prosopis woodland, Argentina	NA	*r* = 0.174^a^, ^b^
5ii	Tropical forest, Costa Rica	Light treatment coding: i (shade),ii (light); Response variable: (lifespan), 2 (biomass); Abundance coding: _a_ (adult basal area), _b_ (adult stem density)	*r*_i1a_ = −0.210, *r*_i1b_ = 0.285^a^, *r*_i1_ = 0.038^b^; *r*_i2a_ = −0.535, *r*_i2b_ = −0.596; *r*_ii1a_ = −0.373, *r*_ii1b_ = 0.254, *r*_ii2a_ = −0.097, *r*_ii2b_ = 0.528^a^, *r*_ii2_ = 0.215^b^
6ii	Temperate forest, Michigan, USA	Light treatment coding: i (shade), ii (light); Response variable: (survival), 2 (growth rate), 3 (combined); Abundance coding: _a_ (adult basal area), _b_ (adult stem density)	*r*_i1a_ = 0.727, *r*_i1b_ = 0.841^a^, *r*_i1_ = 0.784^b^; *r*_i2a_ = −0.483, *r*_i2b_ = −0.743; *r*_i3a_ = 0.178, *r*_i3b_ = 0.007; *r*_ii1a_ = 0.408, *r*_ii1b_ = 0.484; *r*_ii2a_ = 0.548, *r*_ii2b_ = 0.454; *r*_ii3a_ = 0.576^a^, *r*_ii3b_ = 0.497, *r*_ii3_ = 0.536^b^
7	Invaded oak savannah, British Columbia, Canada	Abundance coding: _a_ (# plots present), _b_ (cover)	*r*_a_ = 0.590^a^, *r*_b_ = 0.118, *r* = 0.354^b^
8	Grassland, New Zealand	Abundance coding: _a_ (# of plots present), _b_ (dominance)	*r*_a_ = −0.269, *r*_b_ = 0.612^a^, *r* = 0.172^b^
9	Savannah, Tanzania	NA	*r* = 0.663^a^, ^b^
10	Old field, Ontario, Canada	NA	*r* = 0.753^a^, ^b^
11	Semi‐natural grasslands, Germany	NA	*r* = 0.780^a^, ^b^
12	Semi‐natural grasslands, Germany	NA	*r* = −0.097^a^, ^b^
13	Tropical forest, Panama	Abundance coding: _a_ (adult stem density), _b_ (adult basal area)	*r*_a_ = 0.762, *r*_b_ = 0.905^a^, *r* = 0.834^b^

Abbreviation: NA, not applicable.

^a^ The most positive correlation coefficient (*r*) per experiment, providing the most conservative synthesis test of the null hypothesis of no positive abundance‐feedback correlation.

^b^ Average *r* per experiment and more liberal test of the null hypothesis.
